# En-bloc resection including SMV and duodenum in patient of locally advanced colon cancer

**DOI:** 10.1093/jscr/rjac610

**Published:** 2023-01-04

**Authors:** Jeong Hee Han, Byoung Chul Lee, Byeong Gwan Noh, Jae Kyun Park, Jung Bum Choi, Young Mok Park, Hyuk Jae Jung, Hong Jae Jo

**Affiliations:** Department of Surgery, Pusan National University Hospital, 179, Gudeok-ro, Seo-gu, Busan 49241, Korea; Department of Surgery, Pusan National University Hospital, 179, Gudeok-ro, Seo-gu, Busan 49241, Korea; Department of Surgery, Pusan National University Hospital, 179, Gudeok-ro, Seo-gu, Busan 49241, Korea; Department of Surgery, Pusan National University Hospital, 179, Gudeok-ro, Seo-gu, Busan 49241, Korea; Department of Surgery, Pusan National University Hospital, 179, Gudeok-ro, Seo-gu, Busan 49241, Korea; Department of Surgery, Pusan National University Hospital, 179, Gudeok-ro, Seo-gu, Busan 49241, Korea; Department of Surgery, Pusan National University Hospital, 179, Gudeok-ro, Seo-gu, Busan 49241, Korea; Department of Surgery, Pusan National University Hospital, 179, Gudeok-ro, Seo-gu, Busan 49241, Korea

## Abstract

Tumor could directly invade or is adherent to other organs, but superior mesentery vein (SMV) and duodenum invasion are very rare. A 62-year-old woman was diagnosed with abdominal pain for several months. Multiple erythematous brownish skin patches and palpable mass were found at epigastric area. Computed tomography imaging showed focal wall thickening at the transverse colon that invaded to the rectus muscle and anterior abdominal wall. On exploration, we identified tumor invaded or was adherent to the duodenum and superior mesenteric vein and performed en-bloc resection. After surgery, the patient received chemotherapy and was followed up without any recurrence for 16 months. Adhesion and invasion of tumor to surrounding organs can be unexpectedly found during surgery. In our case, we found duodenum and SMV invasion and achieved R0 resection by SMV and duodenum resection, which could improve the patient’s prognosis.

## INTRODUCTION

Locally advanced colon cancer (LACC) is clinically defined as primary colon cancer with direct invasion to the adjacent structures or extensive regional lymph node involvement. Incomplete excision of the tumor and residual tumor in the neighboring organs are risk factors for poor prognosis and recurrence in LACC. En-bloc radical colectomy initially proposed by Bacon still remains controversial and is a challenging surgical matter [1]. Several studies reported that en-bloc resection, including R0 resection, improves survival [2–4]. We report an unusual case of transverse colon cancer with local invasion to duodenum third portion, superior mesenteric vein and anterior abdominal wall.

## CASE REPORT

**Figure 1:**
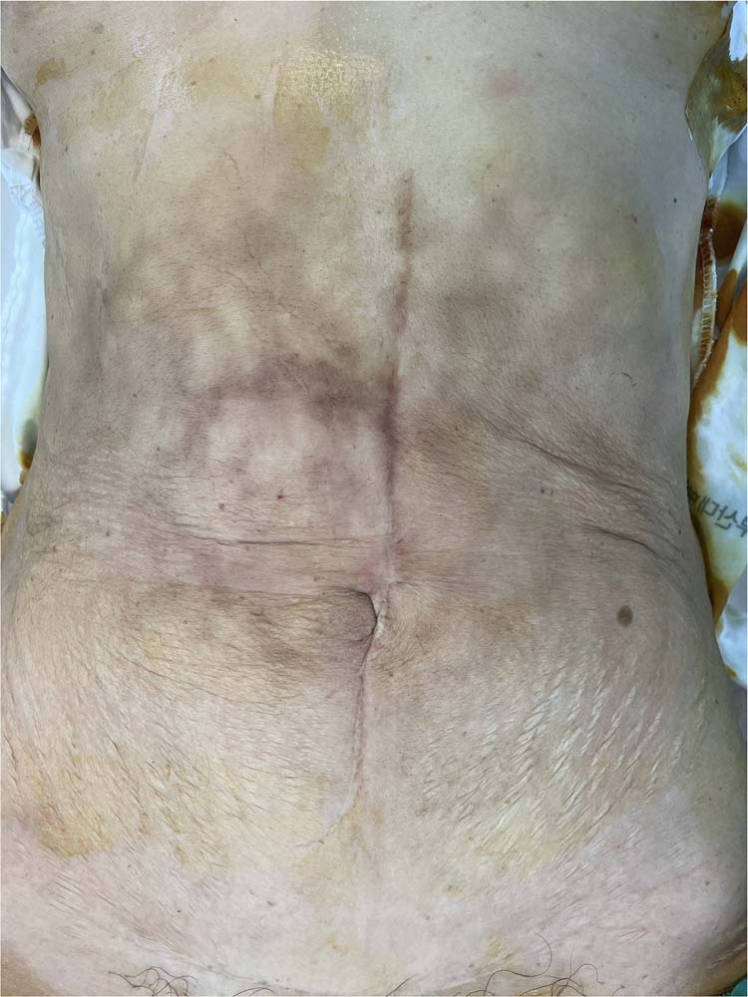
Multiple erythematous brownish skin patches and palpable mass at epigastric area.

A 62-year-old woman was referred to the emergency room with abdominal pain. She had hypertension and had undergone right hemicolectomy due to colon cancer 22 years ago. A physical examination revealed multiple erythematous brownish skin patches and palpable mass at epigastric area (Fig. 1). All routine laboratory investigations were unremarkable, except carcinoembryonic antigen, 15.5 ng/mL.

**Figure 2:**
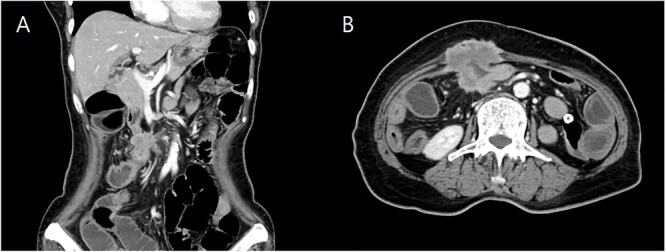
Coronal (**A**) and axial (**B**) computed tomography image of abdomen and pelvis showing transverse colon cancer with direct invasion to the rectus muscle and suspicious invasion of superior mesenteric invasion.

She underwent computed tomography (CT), which showed focal wall thickening at the transverse colon with direct invasion to the rectus muscle and subcutaneous fat layer of the anterior abdominal wall (Fig. 2). The surrounding multiple small lymph nodes were suspected of metastasis. There was another 2-cm tumor lesion in the sigmoid colon. She was diagnosed locally advanced transverse colon cancer and early sigmoid colon cancer after colonoscopy and biopsy.

**Figure 3:**
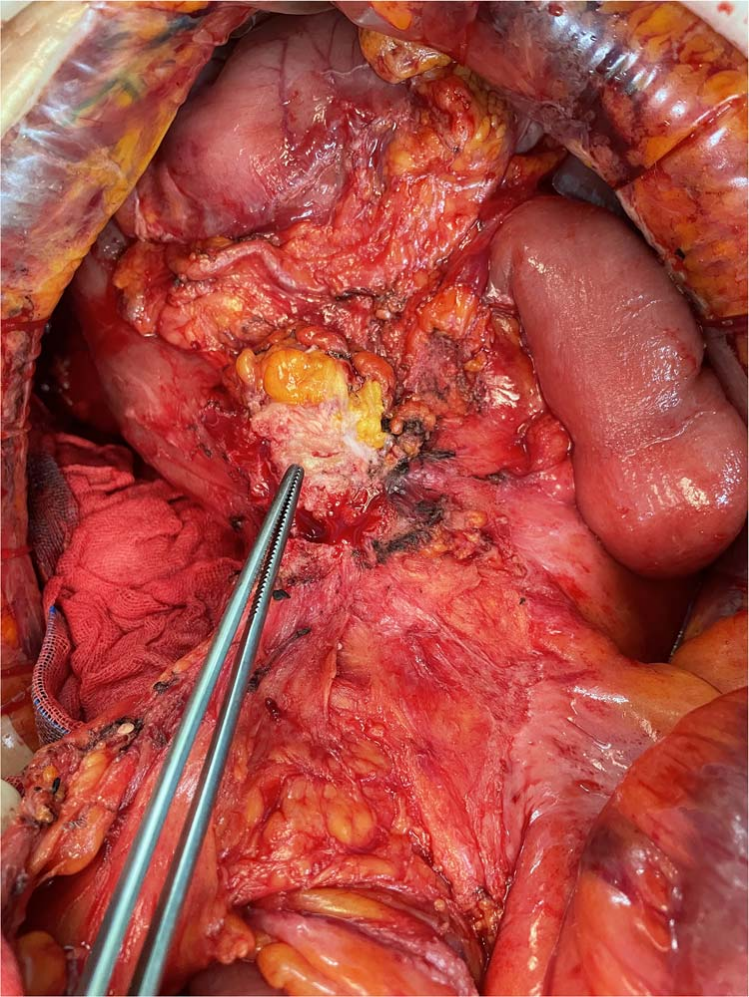
Tumor invaded or was adherent to the duodenum and superior mesenteric vein.

**Figure 4:**
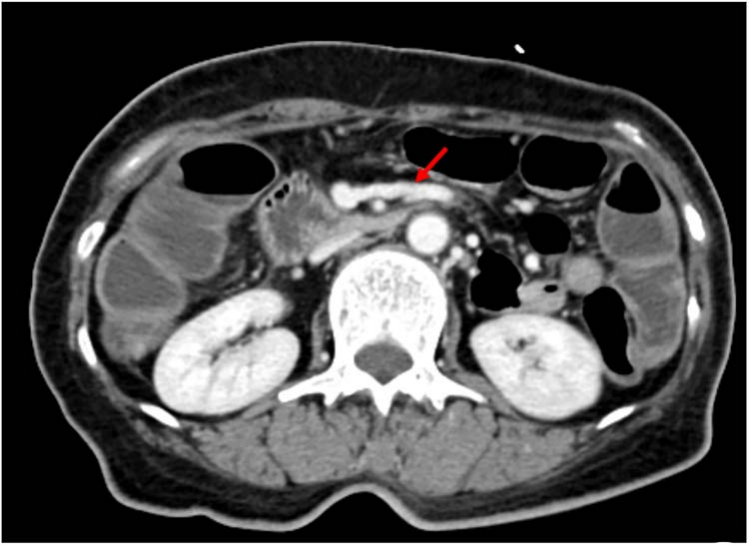
Dilated jejunal first branch of superior mesenteric vein (red arrow).

**Figure 5:**
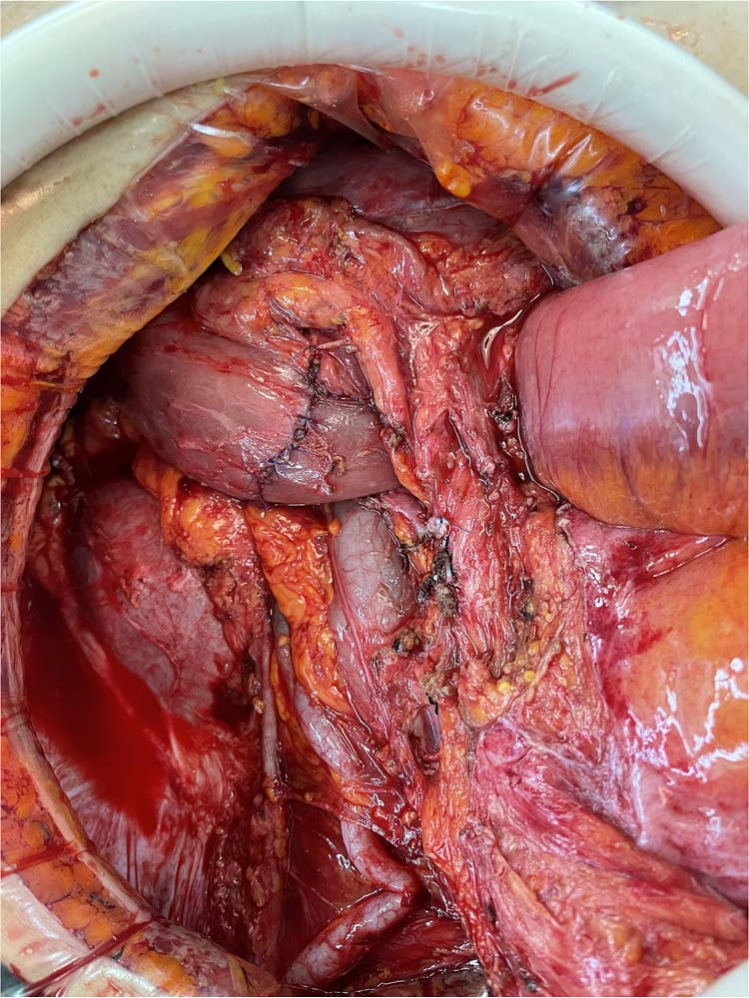
Appearance after en-bloc resection.

We made an incision avoiding the tumor. The patient’s abdominal cavity showed overall adhesion due to previous surgery. We found tumor invading or being adherent to the duodenum and superior mesenteric vein (Fig. 3). We thought that superior mesentery vein (SMV) collapsed due to compression, but it was identified completely occluded due to invasion. We also found that jejunal first branch was very dilated due to obstruction (Fig. 4). We performed completion right hemicolectomy with duodenal segmental resection, SMV resection and anastomosis (Fig. 5). Anterior resection was performed for the accompanying sigmoid colon cancer.

The pathology results revealed a T4bN1b moderately differentiated adenocarcinoma, negative resection margins, and positive lympho-vascular and perineural invasion at transverse colon and a T1N0 moderately differentiated adenocarcinoma, negative resection margins, positive lymphatic invasion at sigmoid colon. The patient’s postoperative course was uneventful and she was discharged on the 23rd post-operative day. After surgery, she received FOLFOX chemotherapy and was being followed up without any recurrence for 16 months.

## DISCUSSION

At the time of diagnosis, 10–20% of patients have LACC, which means that the tumor has penetrated the bowel wall and infiltrated neighboring organs. Historically, patient with an LACC was expected to have a poor prognosis. Recent studies, however, report that their survival can be comparable to that of patients with less advanced tumors if effective treatment is administered [5, 6]. In addition, several studies reported that en-bloc resection, including R0 resection, improve survival [1–4]. Radical tumor excision helps increase survival, although the surgical removal of a locally advanced tumor might be problematic due to organ involvement.

Among the multi-visceral resections for LACC, jejunal and ileal resections are performed most often and duodenum and SMV resection are extremely rarely performed. The peritoneum (35%), abdominal wall (25%), small bowel (other than the duodenum) (16%), omentum (16%), urinary bladder (14%), and ovaries (12%) were the most frequently invaded adjacent structures; invasion of the duodenum (3%) uterus (4%) stomach (4%) retroperitoneum (4%) and Gerota’s fascia (2%) was uncommon [7]. Invasion of major vessels is very rare. Uludag et al. [8] performed SMV resection and anastomosis in only one of their six pancreatic and duodenal invasion cases.

Thanks to the development of CT technology, lymph node and distant organ metastases can be evaluated with high accuracy and the diagnosis of LACC is possible pre-operatively [9]. CT can detect distant organ metastases (particularly liver metastases) and T4 stage at a high rate [10]. However, CT alone may not be able to distinguish direct invasion from an inflammatory reaction. Even during the surgery, distinction between inflammation and cancer is more difficult. Histopathological investigation is the only way to make a conclusive diagnosis [11]. Malignancy was discovered in between 50 and 94% of adhesions detected during the procedure via histological investigation [10, 12]. As a result, intraoperative adhesions should be regarded malignant lesions [13]. Although endoscopy aids in determining the duodenal invasion of right colon malignancies, the duodenal mucosa can be observed as normal only in situations with serosa invasion [11]. In our case, abdominal wall invasion and lymph node metastases were discovered, but adjacent organs particularly SMV or duodenal infiltration were not found, and no duodenal infiltration was detected in endoscopic findings.

Infiltration in surrounding organs can be unexpectedly found during surgery, however, proper resection may not be performed due to the patient’s advanced age and the surgeon’s lack of experience. Without R0 resection, 5-year survival rate ranges from 0 to 23% [10, 12]. However, the 5-year survival rate is between 49 and 61% when en-bloc resection of the colon and surrounding organs is performed [2]. There is no substantial difference between mortality and morbidity even in old age [14]. Therefore, even if adjacent organ infiltration is found during surgery, proper resection should be undertaken.

In conclusion, adhesion and invasion of adjacent organs can be unexpectedly found during locally advanced colorectal cancer surgery. In our case, we found duodenal and SMV invasion during surgery and achieved R0 resection by SMV and duodenal resection, which could improve the patient’s prognosis.
